# Highly-Efficient Plasmon-Enhanced Dye-Sensitized Solar Cells Created by Means of Dry Plasma Reduction

**DOI:** 10.3390/nano6040070

**Published:** 2016-04-14

**Authors:** Van-Duong Dao, Ho-Suk Choi

**Affiliations:** Department of Chemical Engineering, Chungnam National University, 220 Gung-Dong, Yuseong-Gu, Daejeon 305-764, Korea; duongdaovan@cnu.ac.kr or duongdaovan@gmail.com

**Keywords:** plasmonic, dry plasma reduction, Ag nanoparticles, Au nanoparticles, dye-sensitized solar cells

## Abstract

Plasmon-assisted energy conversion is investigated in a comparative study of dye-sensitized solar cells (DSCs) equipped with photo-anodes, which are fabricated by forming gold (Au) and silver (Ag) nanoparticles (NPs) on an fluorine-doped tin oxide (FTO) glass surface by means of dry plasma reduction (DPR) and coating TiO_2_ paste onto the modified FTO glass through a screen printing method. As a result, the FTO/Ag-NPs/TiO_2_ photo-anode showed an enhancement of its photocurrent, whereas the FTO/Au-NPs/TiO_2_ photo-anode showed less photocurrent than even a standard photo-anode fabricated by simply coating TiO_2_ paste onto the modified FTO glass through screen printing. This result stems from the small size and high areal number density of Au-NPs on FTO glass, which prevent the incident light from reaching the TiO_2_ layer.

## 1. Introduction

Dye-sensitized solar cells (DSCs) can achieve comparatively high conversion efficiency levels at a low fabrication cost [1]. In general, a DSC has a working electrode (WE) in the form of a nano-crystalline TiO_2_ electrode modified with a dye and fabricated on a transparent conducting oxide (TCO), an iodide electrolyte solution and a platinum (Pt) counter electrode (CE). The WE has the greatest potential to improve the efficiency of a DSC, with the efficiency defined as the light-harvesting efficiency, due to its monolayer of adsorbed dye molecules [2]. In order to improve the photocurrent, tremendous efforts have been made, including efforts to control the thickness of the TiO_2_ layer [3], synthesize new dyes [4] or use a scattering layer [5]. A major problem, however, is the attachment of less dye, reducing the short-circuit current density [6]. One approach to solve this problem is based on a plasmonic structure, such as silver nanoparticles (Ag-NPs) [6,7], gold nanoparticles (Au-NPs) [8] or gold nanorods [9]. Thus far, plasmonic structures have been prepared by sputtering combined with annealing [6], soaking in a colloidal silver solution for as many as 15 hours [7] or with a plasmonic paste [8,9]. These techniques, however, require toxic chemicals for the synthesis process, high temperatures for annealing, extended times for soaking or a vacuum chamber for sputtering, all indicating that developing an economic continuous process remains a challenge [10].

In order to overcome these process restrictions, we developed a new process of efficiently synthesizing supported metal-NPs by means of dry plasma reduction (DPR) [10,11,12]. More importantly, the developed method can operate at temperatures close to room temperature under atmospheric pressure and without using toxic chemicals. In this communication, we use the DPR method to synthesize Au-NPs and Ag-NPs on fluorine-doped tin oxide (FTO) glass substrates. The main goal of this study is to improve photocurrent generation in DSCs by studying the plasmon enhancement effect. This work is not only for developing a simple strategy of realizing how to improve photovoltaic properties of the DSC using NPs-coated FTO, but also for testing a potential concept of other applications, such as the optical devices, chemical catalysts and biological sensors.

## 2. Results

**Figure 1:**
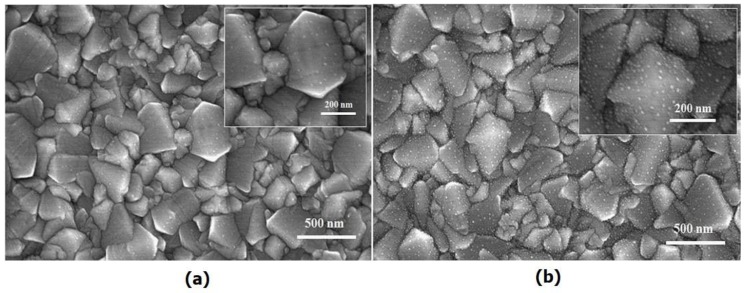
(**a**) High-Resolution Scanning Electron Microscopy (HRSEM) image of Ag-nanoparticles (NPs) on an fluorine-doped tin oxide (FTO) glass substrate; and (**b**) HRSEM image of Au-NPs on an FTO glass substrate.

The overall DPR fabrication process is thoroughly described in our previous study [10]. Figure 1a,b shows the morphologies of the Ag-NPs and Au-NPs on the FTO glass substrates. As can be seen, Ag-NPs and Au-NPs with a few nanometers in size can clearly be observed to be uniformly formed on the FTO glass surfaces without any aggregation via the DPR process. This uniform and dense distribution of NPs provides high optical transmittance in the NPs on the FTO glass. As shown in Figure 1a,b, the Ag-NPs appear larger than the Au-NPs, while the areal number density of the Ag-NPs is lower than that of Au-NPs on the FTO glass. This is also supported by the Transmission Electron Microscopy (TEM) images of the Ag-NPs/Cu grid (Figure S1a, Supplementary Materials) and Au-NPs/Cu grid samples (Figure S1b, Supplementary Materials). Figure S1a shows a TEM image of Ag-NPs immobilized on a Cu grid. The average size of the Ag-NPs was measured and found to be 6–7 nm. Figure S1b presents a TEM image of Au-NPs immobilized on a Cu grid. The average size of the Au-NPs was in the range of 2–7 nm, typically 3 nm and quite small and highly mono-dispersed. Note that the particle sizes have been estimated through the ImageJ program. The average sizes of the Ag-NPs and Au-NPs on the Cu grid are slightly larger than that of the Pt-NPs on the Cu grid [10]. The size difference between the NPs corresponding to the different metal precursors on the Cu grids is likely due to the different electrostatic energy levels of the metal NPs. The physiochemical conversions of HAuCl_4_ and AgNO_3_ into metallic Au and Ag, respectively, were further confirmed by X-ray photoelectron spectroscopy (XPS) (Supplementary Materials, Figures S2 and S3). As shown in Figure S2, the binding energy levels of the Au 4f_7/2_ and Au 4f_5/2_ electrons are 83.9 and 87.7 eV, respectively. This is consistent with Au metal, which indicates that the Au-NPs/FTO sample exists in a metallic state [13]. Additionally, after DPR, the Cl element composition was completely absent in the XPS spectrum (Supplementary Materials, Table S1). Hence, HAuCl_4_ was completely converted into metallic Au through the DPR process. It was also confirmed that Au-NPs were completely crystallized. The binding energy levels of the Ag 3d_5/2_ and Ag 3d_3/2_ electrons are 368.2 and 374.2 eV, respectively (Supplementary Materials, Figure S3), consistent with Ag metal and indicating that the Ag-NPs/FTO samples are in a metallic state [14]. Identical to the Cl element composition in H_2_PtCl_6_ [10] and HAuCl_4_, the N element composition in AgNO_3_ was also completely absent after DPR (Supplementary Materials, Table S2).

**Figure 2:**
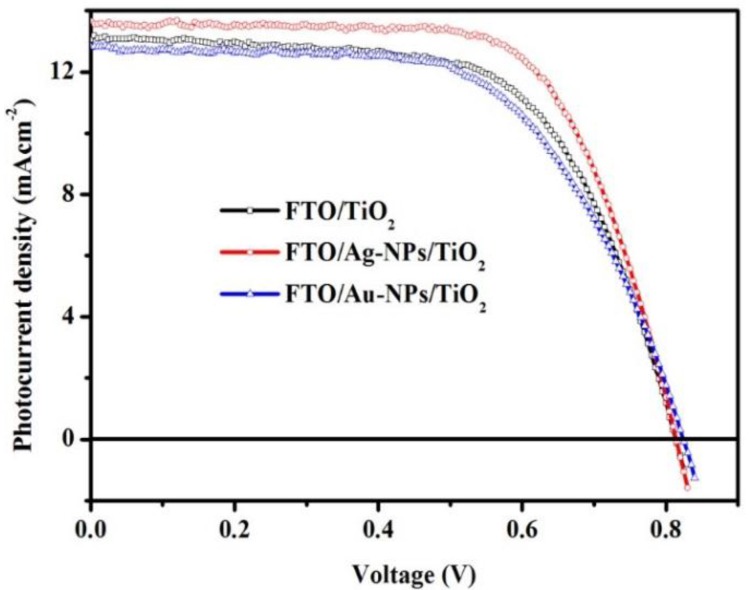
Current-voltage characteristics of three dye-sensitized solar cells (DSCs) equipped with different working electrodes.

**Figure 3:**
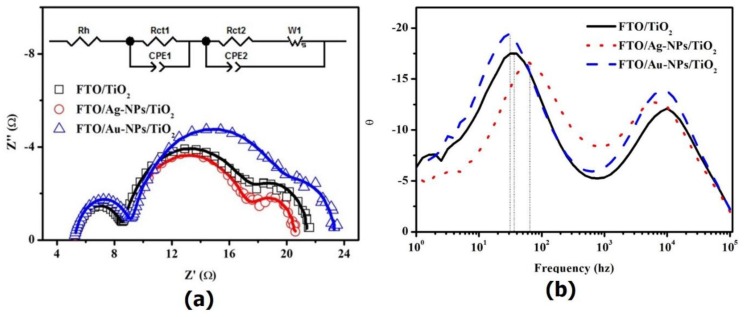
(**a**) Nyquist plots of three DSCs equipped with different working electrodes; and (**b**) The electrochemical impedance spectroscopy (EIS) Bode plots of three DSCs equipped with different working electrodes. Z’’: imaginary part of impedance; Z’: real part of impedance; CPE1: the constant phase element at counter electrode/electrolyte interface; CPE2: the constant phase element at working electrode/electrolyte interface; Rh: Ohmic internal resistance; Rct1: charge-transfer resistance at counter electrode/electrolyte interface; Rct2: charge-transfer resistance at working electrode/electrolyte interface; W1: Warburg impedance.

Figure 2 shows a comparison of the performance capabilities of the three DSCs with different working electrodes (WEs). The DSC with FTO/Ag-NPs/TiO_2_ as the WE shows an energy conversion efficiency of 7.49 (±0.14)%, which is better than those of the DSCs with the FTO/TiO_2_ and FTO/Ag-NPs/TiO_2_ WEs, videlicet 6.54 (±0.15)% and 6.27 (±0.11)%, respectively, as listed in Table 1. Regardless of the illumination condition, the open-circuit voltages, *V*_oc_, of the DSCs with FTO/Ag-NPs/TiO_2_ and FTO/Au-NPs/TiO_2_ as the WEs are slightly higher than that of the DSC with FTO/TiO_2_ as the WE. This phenomenon is understood by considering that the Ag-NPs and Au-NPs are linked together with titania, causing a shift of the conduction band edge of titania to the negative side [15] and further increasing the gap between the Fermi level of the photoelectrode and the redox potential under illumination, which is the definition of photovoltage [16]. The short-circuit current, *J*_sc_, of the DSC with the FTO/Ag-NPs/TiO_2_ WE, however, is slightly higher than that of the DSC with FTO/TiO_2_ as the WE due to the low electron transfer at the TiO_2_/dye/electrolyte interface [9], which was confirmed again by incident photon-to-current efficiency (IPCE) measurements and the electrochemical impedance spectroscopy (EIS) results below. In contrast, the *J*_sc_ of the DSC with FTO/TiO_2_ as the WE is slightly higher than that of the DSC with FTO/Au-NPs/TiO_2_ as the WE due to the higher Schottky barrier in the interfacial region of Au (5.1–5.5 eV) and TiO_2_ (4.0 eV), with a slight difference between the work function of Ag (4.12 eV) and the electron affinity of TiO_2_ (4.0 eV). There were some conflicts between our results and those by Lin *et al.* [6] for FTO/Ag-NPs/TiO_2_ and by Zhang *et al.* [8] for FTO/Au-NPs/TiO_2_. The differences in the NP size and the dense distribution were used to explain these phenomena. Indeed, the larger size and highly dense distribution of Ag-NPs mean that they could not preferentially attach to specific sites on the TiO_2_ surface, resulting in a decrease of the catalytic activity. In contrast, the small size and highly dense distribution of Au-NPs on FTO glass would prevent the incident light from reaching the TiO_2_ layer. The fill factor (FF) of the DSC with the FTO/Ag-NPs/TiO_2_ WE (67.60% ± 0.68%) is higher than those of the DSCs with the FTO/TiO_2_ WE (61.71% ± 1.16%) and the FTO/Au-NPs/TiO_2_ WE (60.60% ± 1.02%) due to the low internal resistance of the cell [17]. This becomes clear when examining EIS results, as shown in Figure 3.

Figure 3a presents three Nyquist plots of DSCs with FTO/TiO_2_, FTO/Ag-NPs/TiO_2_ and FTO/Au-NPs/TiO_2_ WEs. Table 2 shows the estimated charge transfer resistance values on both the CE and WE, as well as the constant phase element (CPE) parameters for the interfaces of both the CE and WEs. For the purpose of comparing only the WEs, we fabricated three CEs using the same materials and method. As shown in Table 2, none of the parameters pertaining to the CE interface showed a significant difference with regard to the three DSCs. However, there was a distinct difference in Rct2 among DSCs with different WEs, as shown in Table 2. The Rct2 value of the FTO/Ag-NPs/TiO_2_ sample was low, at 3.93 Ω·cm^2^, while those of the FTO/TiO_2_ and FTO/Au-NPs/TiO_2_ samples were 4.24 Ω·cm^2^ and 5.23 Ω·cm^2^, respectively. For our Au-NPs immobilized on FTO, there is direct contact between the Au and the electrolyte, with the surface of the Au-NPs serving as a recombination site for the photogenerated electrons and triiodide ions, resulting in an increase in the charge transfer resistance in comparison to the FTO/TiO_2_ WE [8]. It is known that a lower Rct2 of a cell means faster redox kinetics of I_3_^−^/I^−^ pairs at the photoanode/dye/electrolyte interface [18]. Hence, the electron transfer from the electrolyte to the oxidized dye became rapid, suggesting an efficient injection of electrons to TiO_2_ and transfer across the film to the outer circuit, reducing the rate of electron recombination [18]. This positive factor results in a high IPCE and higher power conversion efficiency. Furthermore, the reduction of the total internal resistance due to the decrease in Rct2 is the cause of the increase in the fill factor of the DSCs [17], which confirms the increase in the conversion efficiency, as previously shown.

For more information about the electron recombination lifetime of the photo-injected electrons during the photovoltaic process (τ_e_ = 1/2π*f*_max_; *f*_max_ is the maximum frequency of the peaks in the intermediate frequency region of EIS [19,20]), EIS Bode plots were devised. We found that the highest and lowest *f*_max_ values were obtained with DSCs based on FTO/Ag-NPs/TiO_2_ and FTO/Au-NPs/TiO_2_ WEs, respectively. Therefore, the shortest and longest τ_e_ distances were obtained for DSCs based on FTO/Ag-NPs/TiO_2_ and FTO/Au-NPs/TiO_2_ WEs, respectively. It is well known that a longer τ_e_ indicates greater effective inhibition of electron recombination during the electron transfer process across WE films. Furthermore, a shorter τ_e_ results in higher photoelectron collection efficiency at the FTO substrate and higher *J*_sc_ values of devices. These results are in good agreement with the current-voltage (*J-V*) results.

**Figure 4:**
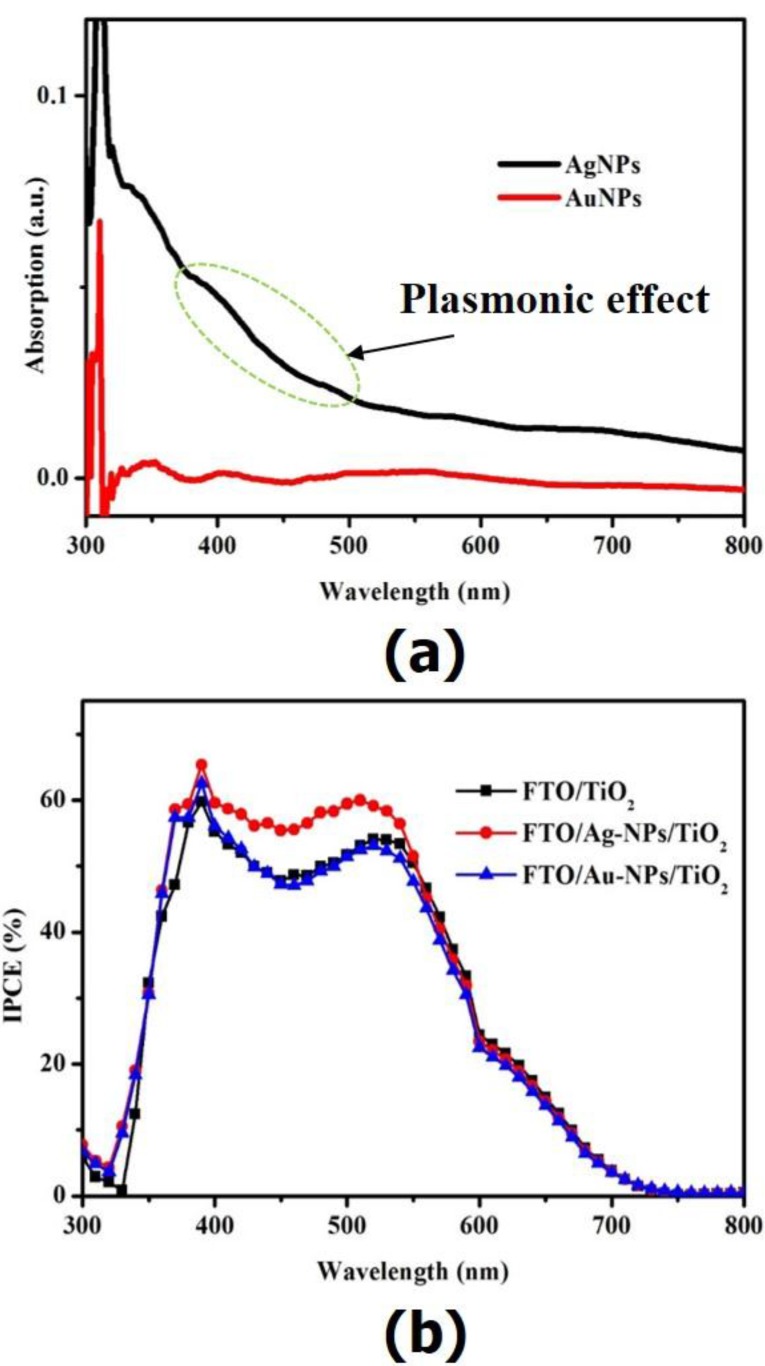
(**a**) Absorption spectra of AuNPs and AgNPs; (**b**) Incident photon-to-current efficiency (IPCE) spectra of DSCs based on different working electrodes. a.u.: arbitrary unit.

Figure 4a shows the absorption spectra of only NPs layer-coated FTO glass substrate with respect to wavelength. As can be observed, the absorption spectra of AuNPs-coated FTO glass substrate becomes almost zero within the range of wavelength from 300 to 800 nm. It was close to the absorption of FTO glass substrate. The results indicate that the plasmonic effect of AuNPs incorporated on FTO glass substrate could be neglected. However, the surface plasmonic absorption of AgNPs was clearly presented in about 400 nm. The absorption peak of the AgNPs/FTO glass was shifted compared to the AgNPs/FTO glass in other studies [6,21] due to the broad size distribution with large AgNPs. The radiant light can be enhanced after being effectively coupled with plasmon absorption in the range of 300–600 nm due to the increase of the optical density around AgNPs [9]. According to that, there were more photons, which was usually absorbed by the dye molecules placed in the vicinity of AgNPs [21,22,23], resulting in the improvement of the device performance.

In order to investigate the effect of plasmonic structure on the enhancement of the photocurrent density of devices, we conducted IPCE measurements. The results are given in Figure 4b. As can be seen, the IPCE of the DSC fabricated with the FTO/Ag-NPs/TiO_2_ WE was higher than those of the DSC based on the FTO/TiO_2_ WE and the FTO/Au-NPs/TiO_2_ WE over the visible wavelength range. The current value calculated from the overlap integral of the IPCE spectrum agrees well with the *J*_sc_ derived from the *J–V* characteristics. It is well-known that the IPCE is composed of the light-harvesting and charge collection efficiencies, the efficiency of the electron injection from the excited dye into the TiO_2_ and the efficiency for the dye regeneration. In this study, identical compounds were used to fabricate all devices, except the WEs. Therefore, the effect of the efficiency for the dye regeneration was excluded in this work. It is reported that the plasmonic enhancement is estimated through the improvement of optical density near the metal surface. Thus, more photons could be harvested by dye located in the vicinity of the NPs, resulting in the increase of IPCE [21,22,23]. As we mentioned above, the plasmon effect of AuNPs/FTO glass substrate was not significant. Thus, IPCE of DSC fabricated on AuNPs/FTO was similar to that of DSC assembled on FTO glass substrate. In contrast, the IPCE of the device based on AgNPs/FTO glass substrate became higher than those of other two DSCs due to the transverse plasmon absorption at around 400 nm. The result is in good accordance to Ultraviolet–visible spectroscopy (UV-Vis) data and *J–V* results.

## 3. Materials and Methods

### 3.1. Materials

H_2_PtCl_6_·*x*H_2_O (≥37.5% Pt basic), AgNO_3_ (>99%) and iso-propyl alcohol (IPA) (99.5%) were obtained from Sigma-Aldrich (St. Louis, MO, USA). HAuCl_4_·3H_2_O was purchased from Aldrich. FTO glass as a conductive transparency electrode was purchased from Solaronix, Aubonne, Switzerland (~8 Ω/square). These substrates were used after cleaning them by sonication in acetone (Fluka). The nonporous TiO_2_ paste and ruthenium-based dye (N719) used in the study were purchased from Solaronix, Aubonne, Switzerland. The dye was adsorbed from a 0.3 mM solution in a mixed solvent of acetonitrile (Sigma-Aldrich) and tert-butyl alcohol (Sigma-Aldrich) (St. Louis, MO, USA) with a volume ratio of 1:1. The electrolyte was a solution of 0.60 M 1-methyl-3-butylimidazolium iodide (Sigma-Aldrich), 0.03 M I_2_ (Sigma-Aldrich), 0.10 M guanidinium thiocyanate (Sigma-Aldrich) and 0.50 M 4-tert-butylpyridine (Sigma-Aldrich) in a mixed solvent of acetonitrile (Sigma-Aldrich) and valeronitrile, with a volume ratio of 85:15.

### 3.2. Synthesis of Au and Ag on FTO Glass Substrates

Two solutions containing 10 mM HAuCl_4_·3H_2_O in IPA and 10 mM AgNO_3_ in IPA were initially prepared. Separately, 8 µL of a precursor solution were deposited onto 2 × 2 cm^2^ specimens of FTO glass, and the solvent was allowed to evaporate at 70 °C for 10 min. The specimens were then reduced using Ar plasma under atmospheric pressure at a power of 150 W, a gas flow rate of 5 litter per minute (lpm), a treatment time of 15 min and a substrate moving speed of 5 mm/s [10]. The morphologies of the Ag-NPs and Au-NPs on the FTO glass substrates were observed by high-resolution scanning electron microscopy (HRSEM). For the TEM measurements, we separately synthesized Ag-NPs and Au-NPs on Cu grids under the same plasma conditions used earlier.

### 3.3. Preparation of the Working Electrodes

Three WEs were prepared on FTO, Ag-NPs/FTO and Au-NPs/FTO glass substrates. The area of TiO_2_ mesoporous layer and that of the TCO were 0.7 × 0.7 cm^2^ and 2 × 2 cm^2^, respectively. These WEs were fabricated through the following procedure. A transparent film of 20-nm TiO_2_ particles (Solaronix, Switzerland) was coated onto the substrates by screen printing (200 T mesh), kept in a clean box for 3 min so that the paste could relax to reduce surface irregularities and then dried for 3 min at 125 °C. This screen-printing procedure (coating, storing and drying) was repeated until the thickness of the working electrode was approximately 12 µm. The electrodes coated with the TiO_2_ paste were gradually heated under an air flow at 325 °C for 5 min, at 375 °C for 5 min, at 450 °C for 15 min and finally at 500 °C for 15 min under ambient conditions [10]. After cooling to 80 °C, the TiO_2_ electrodes were immersed in a 0.3 mM Di-tetrabutylammonium cis-bis(isothiocyanato)bis(2,2’-bipyridyl-4,4’-dicarboxylato)ruthenium(II) (N719) dye solution in a mixture of acetonitrile (Sigma-Aldrich) and tert-butyl alcohol (Aldrich) (volume ratio of 1:1) and kept at room temperature for 24 h to complete the sensitizer uptake process.

### 3.4. Preparation of the Counter Electrodes

Pt CEs were also prepared through DPR, as described in our previous study [10].

### 3.5. Assembly and Measurement of the DSCs

The assembly and characterization of the DSCs were carried out as described in our previous study [10].

## 4. Conclusions

Au-NPs and Ag-NPs were successfully immobilized on FTO glass substrates by means of DPR, with the results showing homogeneous size dispersity. HRSEM and TEM images showed that the areal number density of Au-NPs/FTO was higher than that of Ag-NPs/FTO glass, while Au-NPs/FTO glass was smaller than that of Au-NPs/FTO glass. The DSC with FTO/Ag-NPs/TiO_2_ as the WE demonstrated energy conversion efficiency of 7.49 (±0.14)%, which was better than those of DSCs with FTO/TiO_2_ and FTO/Ag-NPs/TiO_2_ WEs, *viz*. 6.54 (±0.15)% and 6.27 (±0.11)%, respectively.

## Figures and Tables

**Table 1:** Photoelectric performance of the three cells shown in Figure 2. *J*_sc_: The short-circuit current; *V*_oc_: the open-circuit voltages; FF: Fill Factor; η: efficiency; FTO: fluorine-doped tin oxide; NPs: nanoparticles.

Working Electrode	*J*_sc_ (mA·cm^−2^)	*V*_oc_ (mV)	FF (%)	η (%)
FTO/TiO_2_	12.83 ± 0.21	820.00 ± 5.00	61.71 ± 1.16	6.54 ± 0.15
FTO/Ag-NPs/TiO_2_	13.49 ± 0.16	821.66 ± 11.5	67.60 ± 0.68	7.49 ± 0.14
FTO/Au-NPs/TiO_2_	12.34 ± 0.67	826.67 ± 7.63	60.60 ± 1.02	6.27 ± 0.11

**Table 2:** Impedance parameters of three DSCs with FTO/TiO_2_, FTO/Ag-NPs/TiO_2_ and FTO/Au-NPs/TiO_2_ working electrodes (WEs), as estimated from the impedance spectra and equivalent circuit shown in Figure 3a. Rh: Ohmic internal resistance; Rct1: charge-transfer resistance at counter electrode/electrolyte interface; Rct2: charge-transfer resistance at working electrode/electrolyte interface; W1: Warburg impedance; CPE1: the constant phase element at counter electrode/electrolyte interface; CPE2: the constant phase element at working electrode/electrolyte interface. Note that CPE = (CPE-T)^−1^(*jw*)^−(CPE-P)^, in which *j*^2^ = −1, *w* = frequency, CPE-T and CPE-P are frequency-independent parameters of the CPE. *W*s = *R* × tanh([*j* × *T* × *w*]*^P^*)/(*j* × *T* × *w*)*^P^*, where *R* is real part of impedance; *T* is the diffusion interpretation of Warburg element and it is given by *T* = *L*^2^/*D* in which *L* is effective diffusion thickness and *D* is diffusion coefficient of particles; *P* = 0.5.

Working Electrode	Rh (Ω·cm^2^)	Rct1 (Ω·cm^2^)	CPE1-T (µF·cm^−2^)	CPE1-P	Rct2 (Ω·cm^2^)	*W*s			CPE2-T (µF·cm^−2^)	CPE2-P
						*R*	*T*	*P*		
FTO/TiO_2_	2.59	1.81	35.5	0.90	4.24	2.08	0.42	0.5	2410	0.90
FTO/Ag-NPs/TiO_2_	2.58	1.85	36.9	0.89	3.93	1.73	0.43	0.5	2470	0.90
FTO/Au-NPs/TiO_2_	2.56	1.89	36.1	0.90	5.23	1.74	0.40	0.5	2450	0.90

## Supplementary Materials

The following are available online at http://www.mdpi.com/2079-4991/6/4/70/s1.

## Abbreviations

The following abbreviations are used in this manuscript:

DSC: dye-sensitized solar cell
NPs: nanoparticles
FTO: fluorine-doped tin oxide
TCO: transparent conducting oxide
CE: counter electrode
WE: working electrode
DPR: dry plasma reduction
IPA: iso-propyl alcohol
EIS: electrochemical impedance spectroscopy
IPCE: incident photon-to-current efficiency
