# Supplementary material for: Highly-Efficient Plasmon-Enhanced Dye-Sensitized Solar Cells Created by Means of Dry Plasma Reduction
Source: Nanomaterials (Basel). 2016 Apr 14;6(4):70. doi: 10.3390/nano6040070 (PMC5302568; doi:10.3390/nano6040070)
Supplement: Supplementary file 1 [file nanomaterials-06-00070-s001.pdf]

Supplementary Materials: Highly-Efficient Plasmon-Enhanced Dye-Sensitized Solar Cells Created by Means of Dry Plasma Reduction

Van-Duong Dao and Ho-Suk Choi

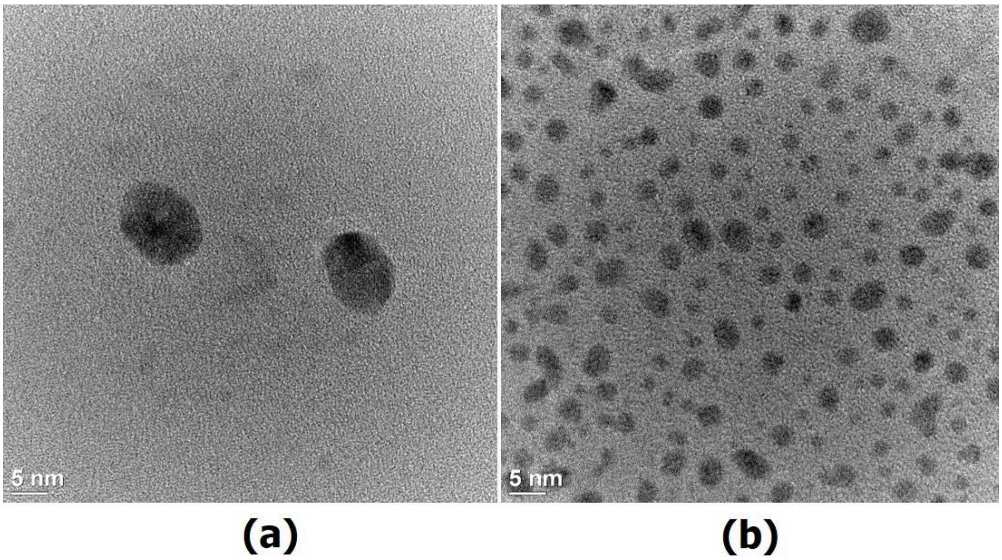

Figure S1. (**a**) Transmission electron microscopy (TEM) images of Ag nanoparticles (NPs) and (**b**) Au NPs on a Cu grid as synthesized by means of dry plasma reduction.

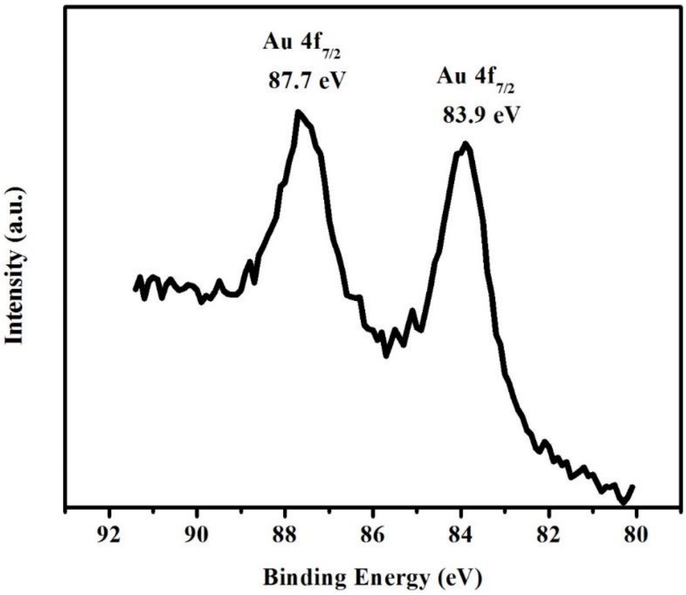

Figure S2. X-ray photoelectron spectroscopy (XPS) spectra of Au nanoparticles on a fluorine-doped tin oxide (FTO) glass substrate. a.u.: arbitrary unit.

Table S1. Elemental composition percentages on the fluorine-doped tin oxide (FTO) glass surface obtained from the dry plasma reduction of $HAuCl_4$ on FTO glass.

Elemental Composition (%)			
Sn	O	Au	Cl
27.23	71.6	1.21	0.00

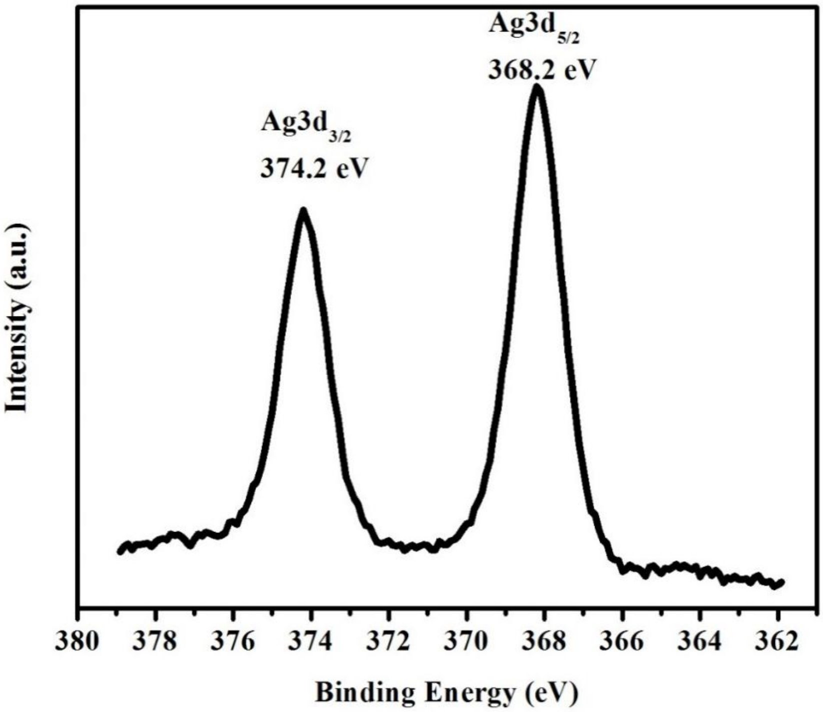

Figure S3. X-ray photoelectron spectroscopy (XPS) spectra of Ag nanoparticles on a FTO glass substrate.

Table S2. Elemental composition percentages on the FTO glass surface obtained from the dry plasma reduction of $AgNO_3$ on FTO glass.

**Elemental Composition (%)**			
Sn	O	Ag	N
26.93	66.49	6.58	0.00

© 2016 by the authors; licensee MDPI, Basel, Switzerland. This article is an open access article distributed under the terms and conditions of the Creative Commons by Attribution (CC-BY) license (http://creativecommons.org/licenses/by/4.0/).
